# Perfectionism and Emotion Regulation in the Study of Suicidal Ideation in Portuguese Young Adults

**DOI:** 10.3390/bs14090846

**Published:** 2024-09-20

**Authors:** Marta Brás, João Antunes, Ana Reis, Cláudia Carmo

**Affiliations:** 1 Psychology Research Centre (CIP), Rua de Santa Marta, 47-3º, 1169-023 Lisbon, Portugal; jpantunes@ualg.pt (J.A.); cgcarmo@ualg.pt (C.C.); 2 Faculty of Human and Social Sciences, University of Algarve, Campus de Gambelas, 8005-139 Faro, Portugal; a57495@ualg.pt

**Keywords:** suicidal ideation, perfectionism, emotion regulation, young adults, Portugal

## Abstract

Suicide is a serious public health problem worldwide, being the culmination of a process that normally begins with suicidal ideation. Therefore, it is important to assess suicidal ideation and know its risk factors. The association between perfectionism and suicidal ideation has been widely debated in the literature. However, knowledge about the role of emotion regulation in this relationship is scarce. The main objective of this investigation was thus to study the role of emotion regulation in the relationship between perfectionism and suicidal ideation in young adults. A sample of 224 Portuguese young adults was recruited through an online form which assessed suicidal ideation, perfectionism, and emotion regulation. The results showed a positive relationship between suicidal ideation and emotion regulation difficulties. There was also a positive association between emotion regulation difficulties and perfectionism, especially regarding the strategies and dimensions of maladaptive perfectionism. The relationship between perfectionism and suicidal ideation was fully mediated by emotion regulation difficulties. Increases in emotion regulation difficulties from increased perfectionism could contribute decisively to increasing the risk of suicidal ideation. Thus, the assessment of perfectionism and emotion regulation difficulties can promote the prevention and psychological interventions for suicidal behavior.

## 1. Introduction

Suicide is a voluntary act to end one’s own life [1] and represents a global public health epidemic, responsible for approximately 700,000 deaths per year [2]. In Portugal, according to the latest data available, in the year 2021, the mortality rate from intentionally self-induced injuries (e.g., suicidal behavior) was 9 per 100,000 inhabitants [3], and this phenomenon is estimated to be the fourth cause of death worldwide among individuals aged between 15 and 29 years [2].

Suicidal behavior consists of a complex and multifactorial process, which encompasses a continuum spectrum of phenomena from thinking about dying to the accomplished act of suicide and may include self-injurious behavior and suicide attempts during this process [4]. Suicidal ideation includes any thoughts associated with the act of ending one’s own life, including both an occasional thought of death or a recurring intention and plan to cause one’s death. Suicidal ideation is therefore a precursor to suicide, and greater attention to its causes is essential to improving the prevention and intervention efforts in this field [5].

The issue of suicidal ideation among suicide cases in Portugal in noticeably pernicious, with one study finding that suicidal ideation was present in 70.2% of suicide cases among studied males [6]. Still, despite the growing evidence that intervening early with at-risk groups is effective [7], there is relatively little research on suicidal ideation in the Portuguese context.

Among Portuguese adolescents, the research seems to indicate that exposure to psychosocial risk seems to affect how life events lead to suicidal ideation [8], highlighting the need to identify and assess whether risk factors might affect young adults similarly. Suicidal ideation among young adults, mainly university students, seems to be associated with social and transgenerational factors [5]. Simultaneously, the trait of perfectionism has been on the rise for decades among college students [9], seemingly generated in part due to some of these latter factors [10,11].

Perfectionism is a transdiagnostic construct present in various psychopathologies related to stress and anxiety, including depression [12]. However, this explanation may be insufficient, given that perfectionism is not always associated with negative outcomes [13]. From a multidimensional perspective, perfectionism integrates adaptive/normative dimensions, like perfectionist efforts, and maladaptive/clinical dimensions, like perfectionist concerns [14]. Some evidence suggests that perfectionist concerns can increase the risk of suicide, while perfectionistic efforts can act as a protective factor [13].

Hewitt and Flett [15] emphasized the intra- and inter-individual aspects of perfectionism, highlighting the following three key components: self-oriented perfectionism, in which the individual places high and possibly unrealistic expectations on themself and seeks perfection; socially prescribed perfectionism, which relates to the belief that others have expectations impossible to fulfil, and that they evaluate the individual too demandingly with punitive judgments and exert pressures related to perfections; and other-oriented perfectionism or externalized perfectionist behavior that expresses a demand for others to be perfect. From the above perspective, SOP and OOP mainly reflect perfectionist efforts and SPP expresses perfectionistic concerns [12].

Again, perfectionism is a risk factor for suicidal ideation [16]. However, it is important to understand the mechanisms by which this process develops. In reviewing emotion regulation difficulties in the context of maladaptive perfectionism, Malivoire et al. [17] identified mindful attention as being a mediator between maladaptive perfectionism and depression, while also pointing out that these results may be indicative that the recognition of internal states can mediate the relationship between depression and perfectionism. Interestingly, this attention to internal states is one of the central constructs in emotion regulation [18], and other emotion regulation capabilities, like self-compassion, are already strongly recognized as mediating the relationship between perfectionism and well-being [19].

Emotion regulation can be broadly understood as the processes that enable one to manage personal emotions in order to function adaptively in emotionally stimulating situations [20]. Effective emotion regulation relates to an individual’s ability to adapt to the demands of the environment through a process of modeling their emotions, associated with the origin or maintenance of positive emotional states and the reduction in negative emotions [21].

Conversely, emotion dysregulation refers to difficulties in identifying, accepting, and re-adjusting the immediate emotional and situational context, leading to a general incapacity to deal adaptively with one’s own emotions, as well as intolerance and sensitivity to emotional suffering and the need to avoid negative emotions [18]. One possible avenue to deal with and avoid such negative emotional states is through self-harming behavior, which often serves the purpose of allowing a person to regulate their emotional state in the short term but at the cost of negative long-term outcomes, particularly an increased suicide risk [22]. As such, emotion dysregulation represents a transdiagnostic risk factor related to psychopathology, in general [23], and suicide, in particular [24,25].

To date, only one study has investigated the relationship among emotion dysregulation, perfectionism, and suicidal ideation [26], finding that emotion regulation, in general, and access to strategies and emotional clarity, in particular, mediated the relationship between perfectionism and suicidal ideation. Better understanding these variables can be particularly pertinent because emotion dysregulation is already a target of interventions for perfectionists [11] and furthering this area of intervention could lead to reduced suicidal ideation within this population [25,27].

With the present study, we thus aimed to analyze the role of perfectionism and emotion regulation in suicidal ideation in a sample of young adults of Portuguese nationality. Our objectives were as follows: (1) analyze the relationship between emotion regulation difficulties and suicidal ideation; (2) analyze the relationship between perfectionistic strivings and concerns and emotion regulation difficulties; and (3) investigate the mediating effect of emotion regulation difficulties in the relationship between suicidal ideation and perfectionism.

## 2. Materials and Methods

Our study sample was composed of 224 Portuguese young adults; 166 participants (74.1%) were female, and all ranged in age from 18 to 35 (M = 22.71; SD = 3.43) years old. All participants were Portuguese, and most (35.7%) lived in the south of the country. Most were unmarried (92.9%) and were either enrolled in or had finished higher education (62.9%). Regarding psychopathological history, 42 (18.8%) of the young adults had already had at least one diagnosed psychological problem, and 85 (37.9%) had already been treated by a mental health professional. Almost half (46.4%) had felt that life was not worth living, 28 (12.5%) participants had committed self-injurious acts, and 15 (6.7%) had attempted suicide. A sociodemographic questionnaire developed in the context of this research collected information on the participants’ age, gender, nationality, residence district, civil status, educational qualifications, clinical and psychological history, and the occurrence of suicidal behaviors.

The Hewitt Multidimensional Perfectionism Scale (HMPS; Hewitt & Flett [15]; adapted by Soares et al. [28]), in its Portuguese adaptation, has satisfactory internal consistency (α = 0.88) and temporal stability (r = 0.85). The scale consists of 45 items scored on a Likert scale (1 = Strongly Disagree; 7 = Strongly Agree), distinguishing the following three dimensions of perfectionism: Self-Oriented Perfectionism (SOP), Socially Prescribed Perfectionism (SPP), and Other-Oriented Perfectionism (OOP). In the present sample, SOP showed excellent reliability (α = 0.91) and SPP had good reliability (α = 0.87). The HMPS is a dispositional scale and therefore has no timeframe associated with its questions.

The Short Almost Perfect Scale (SAPS; Rice et al. [29], adapted by Tomé et al. [30]) is a brief dispositional measure of perfectionism scored with eight items ranked on a Likert Scale (1 = Strongly Disagree; 7 = Strongly Agree). Half the items pertain to Standards, meaning high expectations for the self, and the other half refer to Discrepancy, particularly between the respondent’s current and ‘ideal’ self. These two subscales map onto perfectionistic strivings and concerns, respectively. Both subscales have shown identical, acceptable reliability (α = 0.70) and, in the present sample, the results for Standards were similar (α = 0.73), while Discrepancy showed improved reliability (α = 0.86).

The Positive and Negative Suicidal Ideation Inventory (PANSI; Osman et al. [31], adapted by Brás et al. [32]) is a 14-item measure of both negative (PANSI-NSI) and positive (PANSI-PI) suicidal ideation, reflecting ideation related to the intent to die and the intent to live, respectively, in the last 2 weeks. Both the PANSI-NSI (α = 0.91, 0.93) and the PANSI-PI (α = 0.80, 0.82) showed good reliability in their original inception. The items are scored on a Likert scale (1 = Strongly Disagree; 7 = Strongly Agree), and it is currently being adapted for the Portuguese population. The present sample’s NSI showed excellent reliability (α = 0.96), and PI showed good reliability (α = 0.80).

The Difficulties in Emotion Regulation Scale (DERS; Gratz & Roemer [18], adapted to Portuguese by Coutinho et al. [33]) contains 36 items scored with a 6-point Likert-type scale (0 = Never; 5 = Almost Always). A total score represents emotion dysregulation, and its six facets of specific emotion regulation difficulties as follows: non-acceptance of emotional responses (non-acceptance), difficulty associated with engaging in objective-directed behaviors (objectives), difficulty in controlling impulses (impulses), limited access to effective emotion regulation strategies (strategies), lack of awareness of emotions (awareness), and difficulty in understanding emotional responses (clarity). The scale has shown to have good reliability when taken as a whole (α = 0.93) and in each subscale (α ≥ 0.80), which was replicated in the Portuguese version (α = 0.92; α ≥ 0.75) and in the present sample (α = 0.96; α ≥ 0.85). The scale is dispositional and therefore invokes no timeframe in its questions.

This study’s data were collected by sharing an online form, in a snowball methodology, on social media platforms (e.g., Facebook, Instagram, Twitter), containing the aforementioned measures, during the first half of 2021. Prior to filling out the form, participants were presented with information on the platform about the study and then asked for their informed consent. Thus, the participants knew the purpose of the research, read the guarantee of compliance with ethical questions to scientific research, and were informed that their data were confidential and used solely for the research. Additionally, the respondents were made aware that their participation was voluntary, with the possibility of refusing or abandoning the study. The inclusion criteria were as follows: (a) aged between 18 and 35 years; (b) Portuguese nationality; and (c) presently living in Portugal.

The study design was entirely cross-sectional. To analyze the relationship between emotion regulation difficulties and perfectionism with suicidal ideation, correlations were investigated. Then, to analyze the mediation effects, multiple simple regressions were used. All the data were analyzed in IBM’s SPSS (v. 29.00). Our initial analysis included running descriptives for all study variables and an assessment of reliability for all measures, followed by correlational analysis, measured in Pearson’s r, and mediation analysis, following Baron and Kenny’s [34] recommendations. For mediation analysis, the Discrepancy subscale of the SAPS was used as the predictor due to its consistent association with suicidal ideation, as opposed to the Standards subscale [29]. Neither HMPS subscale was used because social disconnection seemed to be the main mechanism by which these predict suicidal ideation [35]. All the DERS subscales, along with the total scale, were separately tested, as mediators.

## 3. Results

### 3.1. Descriptive Analysis

Table 1 contains descriptives of all the study variables. Our analysis of means and distribution metrics found no serious issues with any variable regarding the sample’s normality or either ceiling or floor effects.

### 3.2. Correlational Analysis

Table 2 summarizes the correlations between emotion regulation difficulties and suicidal ideation, along with the partial correlations controlling for age and gender. Results were generally unaffected by controlling for the other variables. Regarding the relationship between negative ideation and emotion regulation difficulties, a high and significant positive correlation was observed, as well as a positive correlation with all subscales. Regarding the DERS factors, strategies and impulses’ strong and significant correlations (r = 0.58; *p* < 0.001; r = 0.50; *p* < 0.001, respectively) stood out. For the remaining dimensions, moderate and significant associations are observable. These values suggest that subjects with higher levels of negative thinking tended to reveal emotion dysregulation.

Regarding the relationship between positive ideation and total scales on the DERS, a high negative correlation (r = −0.55; *p* < 0.001) was found, as well as a negative correlation on all subscales. Only the strategies dimension had a moderately strong and significant association (r = −0.56; *p* < 0.001). Thus, the individuals with higher levels of positive thinking tended to show less difficulty in emotion regulation.

Table 3 shows the results from our correlation analyses between perfectionism and emotion dysregulation. An overall pattern emerged among both scales, where the maladaptive element of perfectionism showed a strong and statistically significative correlation with all emotion regulation difficulties, while the adaptive dimension showed much weaker, often non-significant, correlations with emotion regulation difficulties. Also, the SAPS’s Standards dimension showed negative correlations with some DERS subscales.

### 3.3. Mediation Analysis

Before analyzing mediation, a simple linear regression was employed to analyze the existence of a linear relationship between perfectionism, as measured by the total SAPS score, and suicidal ideation. The results demonstrated a significant effect of perfectionism on suicidal ideation (β = 0.21; *p* = 0.001), also called the ‘total effect.’ This was followed by a simple linear regression between perfectionism and total emotion regulation difficulties, showing a significant effect of the predictor on the mediating variable (β = 0.39; *p* < 0.001).

**Figure 1:**
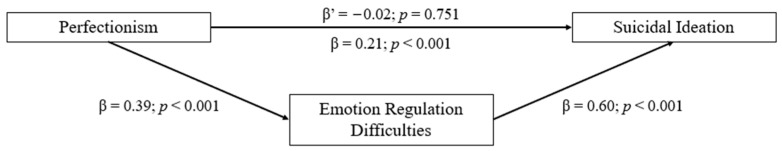
Mediation model of emotion regulation difficulties with perfectionism (SAPS) as predictor and suicidal ideation as outcome.

Next, a multiple regression was carried out, considering perfectionism and the mediating variable (emotion regulation difficulties) as predictors of suicidal ideation. In this sequence, it was possible to conclude that the effect of emotion regulation difficulties on suicidal ideation in the presence of perfectionism was significant (β = 0.60; *p* < 0.001). Additionally, we established with this regression that the relationship between perfectionism and suicidal ideation was non-significant in the presence of the mediating variable (β = −0.02; *p* = 0.751), suggesting total mediation, which was corroborated by the Sobel test that showed a significant indirect effect (Sobel test = 5.38; *p* < 0.001). Figure 1 shows this mediation model (indirect β = 0.23).

Following the previous analysis, each individual emotion regulation difficulty was considered separately as a mediator. Table 4 summarizes the results.

Most measures fully mediated the present relationship, with the following two exceptions: awareness of one’s own emotion, which failed to mediate this relationship, and clarity, which had a partial mediation that only explained 37.7% of the variation found.

## 4. Discussion

With the present research, we aimed to analyze the role of perfectionism and emotion regulation difficulties in suicidal thinking in a sample of young adults of Portuguese nationality. The results in Table 2, regarding the association between emotion regulation difficulties and suicidal ideation, corroborated previous research that indicated that difficulty in regulating emotions is associated with higher levels of negative suicidal ideation (e.g., [24]), possibly due to pre-suicidal behaviors acting as a replacement for more effective emotion regulation strategies [36]. A more in-depth analysis showed that difficulties in selecting effective strategies, controlling impulses, and non-acceptance seemed to be the main risk factors for suicidal ideation, which was also consistent with previous research [37].

The results in Table 3, showing correlational results on the relationship between perfectionism and emotion regulation difficulties, also converged with the previous literature, both in regard to perfectionistic strivings and concerns [38,39]. Interestingly, non-acceptance of emotional states was not correlated with the Discrepancy scores, which is surprising because acceptance and commitment therapy has shown promising results in the treatment of clinical perfectionism [19]. Also, people suffering due to high levels of perfectionism tend to have both high levels of Standards and Discrepancy [29] which, in the context of the present study, could indicate that effectively regulating negative emotions related to perfectionism could be the main differentiator between clinical and non-clinical perfectionism. Although correlational data between perfectionism and suicidal ideation were not collected, the regression results have shown that perfectionistic concerns were predictive of suicidal ideation, as expected [16].

Our mediation results (Figure 1 and Table 4) indicated that emotion regulation difficulties fully mediated the relationship between perfectionism and suicidal ideation. Although these results are congruent with the postulated hypothesis, it was somewhat surprising to see a total mediation effect, particularly because other mediators had been found before (e.g., [40]), so a partial mediation seemed likely. Future research should attempt to integrate multiple mediators simultaneously, perhaps through serial mediation.

We note, however, that ours was not the first study to broach this field of research. Zeifman et al. [26] also investigated the emotion dysregulation’s mediating effect between perfectionism and suicidal ideation. Similar to the current findings, general emotion dysregulation and access to emotion regulation strategies fully mediated this relationship, although Zeifman et al. also found a total mediating effect for emotional clarity, whereas only partial mediation was found in the present study. Additionally, the present data only failed to mediate the relationship with emotional awareness, while Zeifman et al. [26] did not find a mediating effect for the non-acceptance, awareness, impulses, and objectives subscales. Given these data and the fact that the samples were also university students with similar sociodemographic characteristics, the sociocultural contexts of the two countries could induce differences in the concept of perfection and the emotion regulation strategies adopted. Finally, the lack of mediation found for awareness runs contrary to Malivoire et al.’s [17] expectations. Thus, future studies should attempt to replicate our results.

## 5. Conclusions

With the present study, we aimed to explore the role of emotion regulation difficulties in the relationship between perfectionism and suicidal ideation. Our correlational results corroborated the previous literature, separately highlighting the well-established relationship between the relevant constructs.

Although our mediation results did mostly converge with the previous literature (e.g., [26]), some key points were not settled, particularly regarding which emotion regulation difficulties were, in fact, responsible for perfectionism’s effect on suicidal ideation. Fully exploring these findings could have clinical implications by helping stakeholders develop more targeted interventions, especially considering the evidence that supports the targeting of emotion regulation difficulties in perfectionism [17].

This study was mostly limited in how it collected data. Our use of the snowball methodology meant that participants could have self-selected based on their interest in the topic, introducing bias in the results. Additionally, the length of the protocol could raise some issues, as exhaustion can always bias the respondents’ answers.

Future investigators might also focus on capturing comparative data between the clinical and non-clinical samples and perhaps attempt to incorporate the existing data into an intervention study. Additionally, there is a need to develop more complex models that integrate other variables, like parental attachment, which is predictive of various types of perfectionism [41], different emotion regulation difficulties [42], and suicidal ideation [43], suggesting a possible serial mediation model.

## Figures and Tables

**Table 1:** Descriptive statistics for perfectionism, suicidal ideation, and emotion regulation difficulties.

Variable	Mean (SD)	Minimum	Maximum	Skewness (S.E.)	Kurtosis (S.E.)
SAPS ^a^	37.36 (7.67)	17	56	−0.02 (0.16)	−0.09 (0.32)
Standards	21.30 (4.32)	8	28	−0.60 (0.16)	−0.04 (0.32)
Discrepancy	16.06 (6.11)	4	28	0.08 (0.16)	−0.79 (0.32)
HMPS ^b^	128.51 (26.99)	63	217	−0.08 (0.16)	0.16 (0.32)
SOP	82.65 (18.74)	29	125	−0.36 (0.16)	−0.08 (0.32)
SPP	45.87 (13.91)	17	92	0.34 (0.16)	−0.22 (0.32)
DERS ^c^	91.01 (28.05)	40	168	0.39 (0.16)	−0.44 (0.32)
Strategies	19.80 (8.30)	8	40	0.50 (0.16)	−0.54 (0.32)
Non-Acceptance	15.20 (7.30)	6	30	0.42 (0.16)	−0.92 (0.32)
Awareness	13.10 (4.24)	6	25	0.44 (0.16)	−0.03 (0.32)
Impulses	14.48 (6.39)	6	30	0.67 (0.16)	−0.36 (0.32)
Objectives	16.29 (5.25)	5	25	−0.19 (0.16)	−0.70 (0.32)
Clarity	12.14 (4.54)	5	24	0.36 (0.16)	−0.57 (0.32)
Negative Suicidal Ideation	1.53 (0.98)	1	5	2.01 (0.16)	3.04 (0.32)
Positive Suicidal Ideation	3.66 (0.72)	1	5	−0.56 (0.16)	0.36 (0.32)

Note. ^a^ SAPS: the Short Almost Perfect Scale; ^b^ HMPS: Hewitt’s Multidimensional Perfectionism Scale; ^c^ the Difficulties in Emotion Regulation Scale.

**Table 2:** Full and partial correlations between DERS and PANSI subscale scores.

	Full Correlation		Partial Correlations	
	Negative Ideation	Positive Ideation	Negative Ideation	Positive Ideation
DERS ^a^ (total)	0.59 ***	−0.55 ***	0.59 ***	−0.54 ***
Strategies	0.58 ***	−0.56 ***	0.58 ***	−0.56 ***
Non-Acceptance	0.45 ***	−0.36 ***	0.44 ***	−0.35 ***
Awareness	0.34 ***	−0.30 ***	0.34 ***	−0.30 ***
Impulses	0.50 ***	−0.47 ***	0.50 ***	−0.46 ***
Objectives	0.39 ***	−0.39 ***	0.39 ***	−0.36 ***
Clarity	0.38 ***	−0.41 ***	0.38 ***	−0.39 ***

Note. ^a^ The Difficulties in Emotion Regulation Scale; *** *p* < 0.001.

**Table 3:** Results of correlational analysis between HMPS, SAPS, and DERS scores.

	SAPS	Standards	Discrepancy	SOP	SPP
SAPS ^a^	-				
Standards	0.61 ***	-			
Discrepancy	0.83 ***	0.06	-		
HMPS ^b^					
SOP ^c^	0.54 ***	0.58 ***	0.27 ***	-	
SPP ^d^	0.47 ***	−0.03	0.60 ***	0.35 ***	-
DERS ^e^	0.39 ***	−0.14 *	0.58 ***	0.17 *	0.58 ***
Strategies	0.37 ***	−0.15 *	0.57 ***	0.17 *	0.59 ***
Non-Acceptance	0.41 ***	0.04	0.49 ***	0.25 ***	0.45 ***
Awareness	0.09	−0.19 **	0.25 ***	0.02	0.28 ***
Impulses	0.29 ***	−0.12	0.45 ***	0.11	0.48 ***
Objectives	0.30 ***	−0.05	0.41 ***	0.12	0.35 ***
Clarity	0.23 ***	−0.21 **	0.44 ***	0.02	0.45 ***

Note. ^a^ SAPS: the Short Almost Perfect Scale; ^b^ HMPS: the Hewitt Multidimensional Perfectionism Scale; ^c^ SOP: Self-Oriented Perfectionism; ^d^ SPP: Socially Prescribed Perfectionism; ^e^ the Difficulties in Emotion Regulation Scale; * *p* < 0.05; ** *p* < 0.01; *** *p* < 0.001.

**Table 4:** Mediation models for each emotion regulation difficulty with perfectionism as a predictor and suicidal ideation as the outcome.

Mediator	Total Effect	Direct Effect	Indirect Effect	Mediation
Strategies	0.21 **	−0.01	0.22 ***	Full
Non-Acceptance	0.21 **	0.04	0.17 ***	Full
Awareness	0.21 **	0.18 **	0.03	None
Impulses	0.21 **	0.07	0.14 ***	Full
Objectives	0.21 **	0.10	0.11 ***	Full
Clarity	0.21 **	0.13 *	0.08 **	Partial (37.7%)

* *p* < 0.05; ** *p* < 0.01; *** *p* < 0.001.

## Data Availability

The data can be made available for consultation upon request from the corresponding author.
